# Nano-FTIR spectroscopy reveals SiO_2_ densification within fs-laser induced nanogratings†

**DOI:** 10.1039/d4na00409d

**Published:** 2024-08-06

**Authors:** Nadezhda Shchedrina, Gergely Nemeth, Ferenc Borondics, Nadege Ollier, Matthieu Lancry

**Affiliations:** a Institut de Chimie Moléculaire et des Matériaux d'Orsay (ICMMO), Université Paris-Saclay, CNRS Bât. 410 91405 Orsay France nadezhda.shchedrina@universite-paris-saclay.fr; b Laboratoire des Solides Irradiés, École Polytechnique, CNRS, CEA/DRF/IRAMIS, Institut Polytechnique de Paris 91128 Palaiseau Cedex France; c SMIS Beamline, SOLEIL Synchrotron L'Orme des Merisiers 91190 Saint Aubin France

## Abstract

This study explores the structural transformations induced by femtosecond (fs) laser inscriptions in glass, with a focus on type II modifications (so-called nanogratings), crucial for advanced optical and photonic technologies. Our novel approach employs scattering-type scanning near-field optical microscopy (s-SNOM) and synchrotron radiation nanoscale Fourier-transform infrared spectroscopy (nano-FTIR) to directly assess the nanoscale structural changes in the laser tracks, potentially offering a comprehensive understanding of the underlying densification mechanisms. The results reveal the first direct nanoscale evidence of densification driven by HP-HT within fs-laser inscribed tracks, characterized by a significant shift of the main infrared (IR) vibrational structural band of silica glass. It reveals moreover a complex interplay between type I and type II modifications.

## Abstract 2

This study presents the first direct nanoscale evidence of densification within femtosecond laser-inscribed nanogratings, revealing significant structural changes in silica glass using s-SNOM and synchrotron-based nano-FTIR spectroscopy.

## Graphical abstract

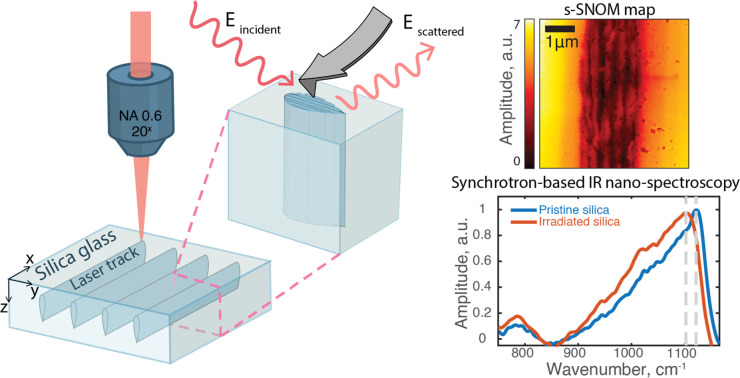

## 1 Introduction

Femtosecond (fs) laser modifications in glass have gained significant attention in material processing, enabling 3D localized modifications at the nanoscale with widespread implications for optical and photonic technologies. These modifications manifest as type I, type II, or type III, dependent on the energy deposition and thus laser processing parameters.^1^ Type I alters the refractive index quite isotropically; type II, referred to as nanogratings, introduces a strong linear birefringence; and type III creates nano/micro-voids with a densified shell.

Among these, type II modifications and underlying nanogratings have become a crucial component in the development of optical and photonic technologies. Owing to their unique linear and circular birefringence^2^ and high thermal stability,^3,4^ nanogratings are used across a broad spectrum of applications, such as 3D optical waveguides,^5^ birefringent devices,^6–8^ advancements in long lifetime optical data storage,^3,9^ temperature sensors,^10,11^ and high-temperature structural health monitoring.^12^

Focusing a fs-laser beam onto glass, such as silica, initiates nonlinear absorption through multiphoton, tunneling, and avalanche ionization mechanisms, resulting in permanent structural modifications.^13^ As the laser intensity surpasses a certain threshold, multiphoton ionization generates a plasma with a high-density electron cloud.^14^ The interference between the incident laser light and light scattered from inhomogeneities of the dielectric constant results in periodic modulations of the electron plasma density and temperature. Such modulations and the evolution of plasma hotspots into elongated nanoplasma regions, driven by local field enhancement, facilitate the emergence of nanogratings.^15^ Oriented perpendicular to the laser's linear polarization, these nanogratings comprise structured layers of oblate nanopores.^16,17^ Despite advances in understanding nanopore formation, the material between these layers, assumed to be densified, remains less understood. Exploring the occurrence of densification and the structural changes within this interlayer material is vital for a full grasp of nanograting mechanisms.

There are several studies focusing on the texture, composition, and properties of the material between fs-laser induced nanogratings. Some experimental investigations have provided indirect evidence of densification within these regions. Following the discovery of nanogratings, studies to understand induced negative uniaxial birefringence revealed significant densification.^18^ Researchers observed an average increase of the refractive index between nonporous layers through the analysis of its directional changes using polarized probe light.

Raman spectroscopy was similarly employed for assessing densification in femtosecond laser-inscribed tracks.^19–24^ Using Raman spectroscopy, Bellouard *et al.*^20^ reported an 8% densification in glass subjected to fs laser processes by drawing comparisons with high-pressure, high-temperature (HPHT) treated samples, indicating a significant alteration in the material's structure. Further studies,^22^ correlating the D2 band intensity and FWHM of the R-band with samples densified by HPHT or HPHT followed by high-energy, high-dose electron irradiation, estimated the post-irradiation glass density to be around 2.25–2.27. However, it should be noted that the D2 band intensity, while increasing linearly with density up to a threshold (around a density of 2.3), may not serve as a definitive density estimator in SiO_2_, as it decreases for densities beyond this point.^25^

Another recent study on fs-laser densification delved into the effects of shock waves generated by two spatially separated focused beams, acting as quasi-simultaneous “pressure-wave emitters”.^23^ This method, using the same approach of silica's Raman signature analysis, estimated the pressure from laser pulses to reach tens of GPa. However, the Raman spectroscopy results, although insightful for densification estimates, reveal the complexity of analyzing the entire laser track due to its composite structure, comprising porous nanolayers and densified interlayers.

Another method to examine the material changes within laser-inscribed tracks involves measuring the volume change and mechanical properties through micro-cantilever deflections.^21,24,26,27^ Type I modifications typically result in volume reduction and material densification, as evidenced by micro-cantilevers moving upwards. Type II modifications cause micro-cantilevers to move downwards due to nanopore expansion. These studies indicate that femtosecond lasers can control a stress-state inversion in bulk fused silica, leading to either stress increase or decrease.^21^ Notably, the material between porous layers shares characteristics with type I modifications, suggesting a denser structure with a higher Young's modulus than pristine silica.^21,27^ Further, while porous layers display a significantly lower Young's modulus, interlayer material exhibits an increased Young's modulus of about 80 GPa^26,27^ suggesting a local densification.

All these findings provide indirect but solid evidence of densification in fs-laser modified regions, however without nanoscale spatial resolution. Despite advances in characterizing fs-laser induced nanogratings, a direct, nanoscale understanding of the densification process remains elusive. Current methods offer a composite view that obscures the magnitude of densification, leaving the specific mechanisms, whether thermal, mechanical, thermo-mechanical, or defect accumulation, quite speculative. Techniques like scanning electronic microscopy (SEM) and atomic force microscopy (AFM) provide nanoscale imaging but fail in revealing the densification process. This gap underscores the need for approaches capable of directly assessing the densification at the nanoscale, which could significantly advance our understanding of the underlying mechanisms. This study utilizes scattering-type scanning near-field optical microscopy (s-SNOM) and synchrotron radiation nanoscale Fourier-transform infrared spectroscopy (nano-FTIR), tools for unveiling the spectral signature of densification at the nanoscale. These methodologies offer unique insights into the densification occurrence and potential underlying mechanisms within type II modifications.

## 2 Experimental details

**Fig. 1:**
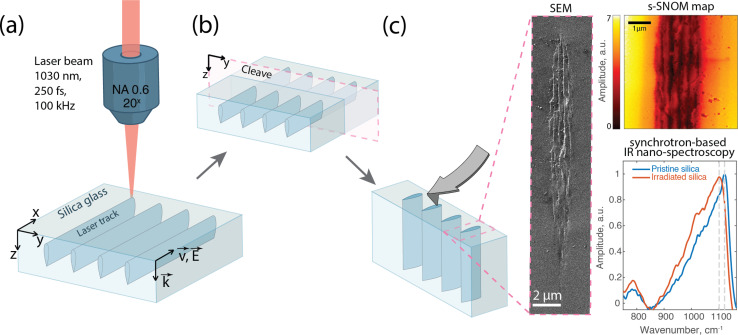
Fabrication and characterization of fs-inscribed laser tracks in silica glass. (a) Laser writing process. (b) Cleaving of the sample for cross-sectional access. (c) Morphological and structural characterization using SEM, s-SNOM amplitude mapping, and synchrotron-based nano-FTIR spectroscopy. Insets show the SEM image and s-SNOM amplitude map illustrating the cross-section of the laser track (200 pulses per micron), as well as nano-FTIR amplitude spectra of both pristine and irradiated areas.

The nanogratings were imprinted in type II silica glass (Suprasil CG, Heraeus) utilizing a fs fiber laser with a wavelength of 1030 nm (Satsuma, Amplitude Systemes Ltd, Pessac, France). The laser emitted pulses at a duration of 250 fs, with a repetition rate of 100 kHz. An aspheric lens with a numerical aperture of 0.6 was used to focus the laser beam at a depth of 200 μm below the surface. The scanning speed varied from 0.005 to 0.8 mm s^−1^ to achieve a range of pulses density values from 1.3 to 20 000 pulses per mm. The laser light was linearly polarized and set in two orientations, parallel (*Xx* configuration) and perpendicular (*Xy*) to the laser writing direction, creating a series of lines with pulse energies exceeding the nanograting threshold (namely 0.3 μJ in our conditions) as schematically described in Fig. 1a.

Post-inscription, the sample was cleaved along the *z*–*y* plane to analyze the cross-section of the laser tracks, see Fig. 1b. Subsequently, the cross-sections were examined using field emission gun scanning electron microscopy (FEG-SEM, ZEISS SUPRA 55 VP, Zeiss, Oberkochen, Germany) to investigate the surface morphology and to construct a detailed map to assist subsequent IR measurements at the synchrotron.

Infrared analyses were conducted at the SMIS beamline of the Synchrotron Soleil in Saint Aubin, France. To characterize the nanostructural modifications (Fig. 1c), we employed scattering-type Scanning Near-field Optical Microscopy (s-SNOM). s-SNOM is a combination of AFM and optical microscopy. It utilizes a metal-coated AFM tip illuminated by focused light. The incident light creates the optical near field that is localized to the apex of the tip. When the tip is brought into close proximity of the sample, the near-field interaction results in scattered light. By measuring both the amplitude and the phase of the scattered light s-SNOM is capable of retrieving the complex optical properties of the sample.^28^

The instrument at SMIS (IR-neaSCOPE, Attocube system AG, Haar, Germany) was used in two different modes. First, taking advantage of the broadband synchrotron radiation nanoscale Fourier-transform infrared spectroscopy (nano-FTIR) was used to collect both infrared near-field amplitude and phase spectra from various sections. This allows identifying optimal wavelengths for subsequent s-SNOM single-wavelength imaging by a built-in quantum cascade laser (QCL). In all the measurements the AFM operated in tapping mode and the optical signal was demodulated at the second harmonic of the tip oscillation frequency.^28^

The main feature of the silica glass infrared spectrum originates from the collective asymmetric stretching vibration of Si–O–Si subunits of its structure.^29^ This infrared active phonon band is located between 900 and 1300 cm^−1^. This strong excitation has its signature both in the amplitude and the phase response. After initial examinations, a wavenumber of 1130 cm^−1^ was chosen from the high wavenumber/frequency edge of the phonon peak for its sensitivity to spectral shifts attributed to fs-laser induced structural changes.

## 3 Results

To investigate the densification of the interlayer material within type II modification, it is important to examine the early stages, the energy threshold of formation during the absence of nanogratings and low birefringence. Therefore, we employed a variation in pulse density, starting from 2 pulses per micron, to capture the initial phase of nanograting development, sometimes related to type X in the literature.^5^ The laser inscription was performed on silica glass, which was prepared as outlined in the experimental section, and subsequently cleaved and examined using FEG-SEM.

**Fig. 2:**
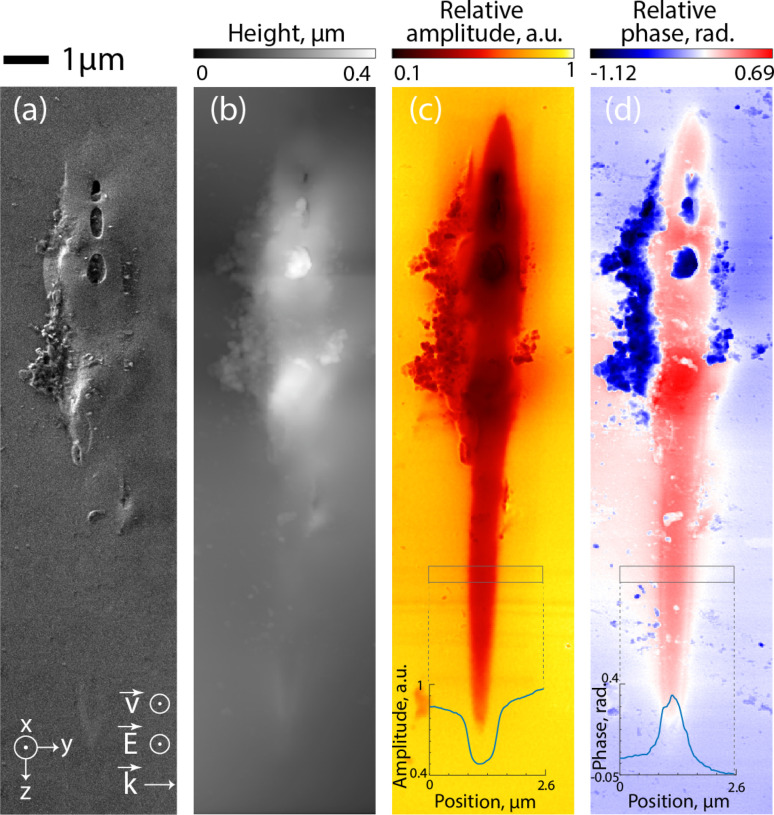
(a) SEM image of a track written with 2 pulses per micron; (b) AFM surface topography; (c) 2D map of near-field scattered amplitude at 1130 cm^−1^; inset: cross-section profile indicating amplitude decrease across the laser track; (d) near-field phase map measured at 1130 cm^−1^.

Fig. 2 shows a laser track inscribed with 2 pulses per micron in an *Xx* (perpendicular) configuration. The SEM image (Fig. 2a) reveals the morphology of the laser track, highlighting topographical changes at the track's head, including the emergence of slightly elongated (along *z*) nanopores. On the other hand, the tail of the track appears flat. This observation is corroborated by the AFM image (Fig. 2b), which similarly depicts topological alterations at the head with no significant changes in the tail, except at the very end. It is important to mention the presence of contaminants along the sides of the laser track, attributable to residual dust due to the cleaving process. This contamination is unrelated to the intrinsic structure of the sample.

Subsequently, we utilized s-SNOM imaging and nano-FTIR spectroscopy to obtain IR maps of the laser tracks. Through near-field amplitude mapping at 1130 cm^−1^, pronounced structural modifications were found as shown in Fig. 2c. Visual examination of the color map distinctly highlighted an extensive area, revealing significant fs-laser induced structural transformations in the silica glass, changes that elude detection by SEM and AFM analyses. Within the irradiated track, the reflectance amplitude at 1130 cm^−1^ was notably reduced in comparison to the unaltered surrounding regions. This observation is additionally confirmed in the cross-sectional profile inset in Fig. 2c. This reduction likely indicates a shift of the IR main band of silica glass to lower wavenumbers, as 1130 cm^−1^ is positioned on the higher-frequency side, or right side, of this band. Similar observations were made in the phase map, which showed significant phase variations within the laser-affected area, typically displaying an increase in the relative phase at 1130 cm^−1^ inside the irradiated volume (see Fig. 2d).

**Fig. 3:**
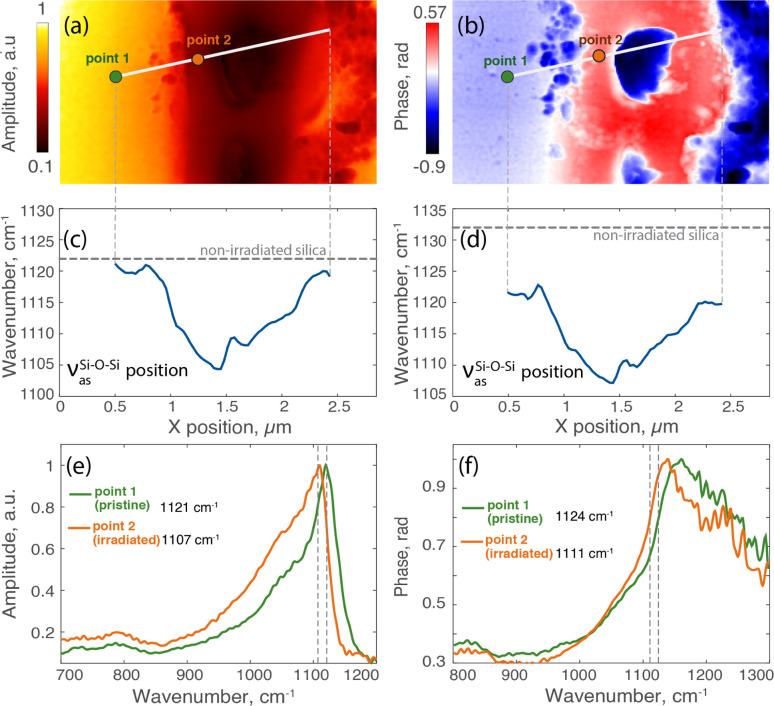
The laser track written with 2 pulses per micron: (a) near-field amplitude map at 1130 cm^−1^; (b) near-field phase map at 1130 cm^−1^; (c) amplitude peak position and (d) phase peak position (measured at 80% of its maximum) along the line profile; (e) synchrotron nano-FTIR amplitude and (f) phase spectra for pristine material and irradiated area inside of the laser track.

Further analysis of the laser track, particularly in the upper area with few nanopores, is presented in Fig. 3a and b, in close-up near-field amplitude and phase maps. We conducted nano-FTIR measurements, along a specified line across this image. Fig. 3e and f display two spectra at points 1 and 2, respectively, outside of the laser track and inside. The spectroscopy data showed significant shifts in peak positions, signaling strong material densification.^30,31^ Notably, the Si–O–Si asymmetric stretching band of silica *ν*^Si−O–Si^_as_, associated with a peak at around 1120 cm^−1^, shifted to lower wavenumbers by more than 14 cm^−1^ in the irradiated region, moving from 1121 to 1107 cm^−1^ (Fig. 3e). In the phase spectra displayed in Fig. 3f, we see similar modifications, consistent with the alterations observed in the amplitude spectra. To accurately track phase spectral changes, we analyzed the wavenumber at 80% of the phase maximum, as the peak itself was a bit noisy, denoting a shift of the low wavenumber shoulder from 1124 to 1111 cm^−1^. Additional amplitude and phase spectra at several other points along this laser track are provided in the ESI in Fig. SI_1.†

The *ν*^Si−O–Si^_as_ shift along line profiles related to amplitude spectra (Fig. 3c) and the one related to the phase measured at 80% of the maximum (Fig. 3d) both reveal a strong and reliable material modification. Starting outside the laser track, the amplitude peak position is stable, representing the unmodified material. Progressing into the laser track, there is a consistent shift of the peak towards lower wavenumbers down to 1103 cm^−1^, with the maximum at the track's center. The slight variation near the center suggests an influence of pores, indicating a small alteration in material density. As the profile extends beyond the track's midpoint, the peak positions rise again, denoting a reduced densification towards the track's periphery. The observed trends in the amplitude peak positions are mirrored in the phase profile, which displays a similar pattern of variation across the laser track. The amplitude and phase spectra used to plot these profiles are provided in ESI Fig. SI_2.†

**Fig. 4:**
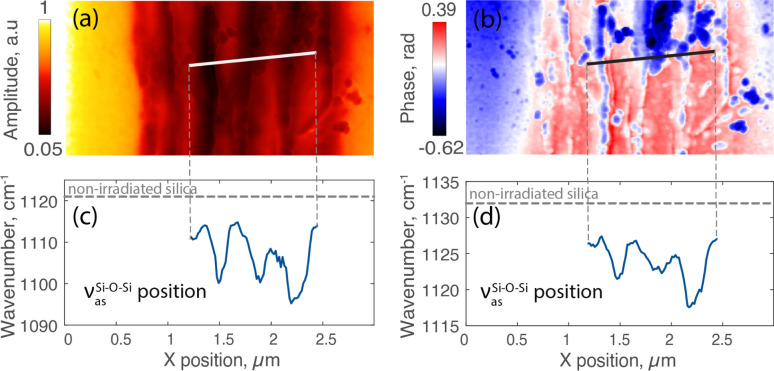
The laser track written with 200 pulses per micron: (a) near-field amplitude map at 1130 cm^−1^; (b) near-field phase map at 1130 cm^−1^; (c) amplitude peak position and (d) phase peak position (measured at 80% of its maximum) along the line profile.

Turning to well-imprinted type II modifications, we investigate a laser track inscribed with 200 pulses per micron in an *Xy* (parallel) configuration, where sufficient pulse density allows for the formation of nanogratings. These are clearly evidenced by the SEM image in the inset of Fig. 1c. Fig. 4a and b provide a detailed view with IR amplitude and phase maps of this laser track. Synchrotron nano-FTIR measurements, conducted along the indicated line in these maps, reveal the nanogratings' structural changes.

Fig. 4c presents the spectral position of the amplitude peak along a cross-sectional line within nanogratings. Starting inside of the laser track at the peak position at 1111 cm^−1^, a pattern of fluctuating peak positions emerges, decreasing and increasing in correspondence with the nanogratings' alternating interlayers (around 1112 cm^−1^) and nanoporous layers. The lowest points (around 1095 cm^−1^) in the graph represent the areas where the nanogratings are densified at the highest degree, near the porous nanolayers. The overall trend appears to oscillate with a periodicity *L*, corresponding to the spacing of the nanogratings of approximately 310 nm. As seen in Fig. 4d, this oscillatory pattern is replicated in the quantitative phase profile revealing a higher phase within interlayers that appear in red on the phase map. The amplitude and phase spectra used to plot these profiles are provided in ESI Fig. SI_3.†

## 4 Discussion

This study advances the current understanding of femtosecond laser-induced modifications in glass, revealing intricate patterns of densification within a laser track. These findings provide the first direct nanoscale evidence of densification within fs-laser inscribed laser tracks, characterized by an interplay of structural modifications such as the formation of porous nanogratings within a background of densified matter. At the early birth of nanogratings *e.g.* within the type X regime, these structural changes are indicative of a mixed modification regime, blending type I and type II processes, and offer a deeper insight into the mechanisms driving the laser-induced modifications observed.

Prior to this work, there was no direct evidence of densification between layers or surrounding nanopores, with most existing results relying on microscale data that average the overall composite nanostructure, where densification has been indirectly inferred based on various hypotheses. However, these investigations have been crucial in providing important estimations and formulating hypotheses that have laid the groundwork for the understanding of fs-laser induced modifications. Previous studies have underscored the utility of Raman spectroscopy and other analytical techniques in elucidating the densification phenomena induced by fs-laser irradiation in silica glass. For instance, pioneering work utilizing scanning thermal microscopy alongside Raman spectroscopy has illuminated the localized densification. These investigations reported an approximate 8% increase in densification and suggested that the densification mechanism involved the development of high pressures.^20^ Further exploration into nanogratings *via* mechanical and optical assessments has supported the hypothesis about the densification of the material. Techniques measuring micro-cantilever deflections have illustrated a change in volume and mechanical properties for type I and type II modifications with tensile stress in type I and compressive stress in type II modifications in bulk fused silica.^21,24,26,27^

This study employs nano-FTIR spectroscopy to delve into the structural nuances of type X and type II modifications within silica glass. IR spectroscopy is a great tool for probing the internal structure of glass, such as the intertetrahedral Si–O–Si asymmetric stretching vibration band near around 1120 cm^−1^.^32^ This spectral feature is instrumental in elucidating the molecular architecture of the glass network, as shifts in this band's position are indicative of alterations in network density, bond angles, and bond lengths. A displacement toward lower wavenumbers denotes a compaction of the network structure, characterized by shorter Si–O bond lengths, a signature of densification.^31,33–36^ The bending mode around 780–800 cm^−1^ is less sensitive than stretching modes due to its intrinsic nature, as the bending vibration is “more internal” and has a lower vibration amplitude, making it less responsive to changes in parameters such as density, stress, or fictive temperature.^37^ This contrasts with the Si–O–Si asymmetric stretching mode response that has been studied and exploited extensively.^31^

In amorphous SiO_2_, a significant shift in the asymmetric stretching vibration band was observed under high-dose (10^13^ cm^−2^) ion bombardment, moving from 1078 cm^−1^ to 1044 cm^−1^.^38^ This shift is attributed to the silica reaching a so-called metamict phase, which is also characterized by an increased presence of 3 and 4-membered rings. However, this does not imply a higher density of the material, as the density of the metamict phase consistently remains at 2.27 and it has been shown that a higher number of 3-membered rings do not always correspond to a higher overall material density.^25^

Another study utilizing FTIR spectroscopy on silica glass demonstrated that the variations in the glass fictive temperature result in slight shifts of *ν*^Si−O–Si^_as_ by only 3 cm^−1^ increase^31,36^ accompanied by a slight densification close to 2% assuming a 400 °C increase in the fictive temperature.

In contrast, a comparable shift to those observed in our findings was demonstrated in another study, where the main band frequency decreases linearly with an increase in density.^30^ Notably, a frequency shift of 30 cm^−1^ was observed for silica subjected to HPHT treatment, resulting in a density increase to 2.55 under densification conditions of 5 GPa pressure and 800 °C temperature. The relationship between IR reflection spectra and density is summarized by the linear dependence:

1 *ν*^Si−O–Si^_as_ = (1332 ± 13) − (95 ± 5)*ρ*,

where *ν*^Si−O–Si^_as_ is the evaluated resonance frequency near 1120 cm^−1^, and *ρ* is the glass density.^30^

In our case, as seen in Fig. 3 and 4, IR spectra exhibit a pronounced shift in this band towards lower wavenumbers. For laser tracks inscribed at a density of 200 pulses per micron, spectral analysis reveals a shift from 1121 cm^−1^ in unirradiated silica to a minimum of 1095 cm^−1^ inside the laser track close to a nanoporous layer, with the interlayer material exhibiting values ranging around 1112 cm^−1^. Consequently, this represents a shift of up to 26 cm^−1^ near within these nanogratings. Using (eqn (1)), we can estimate the laser irradiated silica's density to be 2.495 ± 0.190 for these highly densified regions while the interlayer material correlates with a density of 2.316 ± 0.183. Similarly, for the laser track inscribed with 2 pulses per micron, the minimum peak position in the middle of the track is 1104 cm^−1^, corresponding to a density of 2.400 ± 0.186. The magnitude of these shifts underscores the clear evidence of the densification process not only between nanolayers but all along the laser track and likely around nanopores themselves.

On one hand, our study reveals a complex interplay between type I and type II modifications. At a low pulse density of 2 pulses per micron, we observe the early stages of nanograting formation (so-called type X nanopores) within a surrounding densified matrix, indicative of the onset of the nanocavitation process but within a type I-like background or “bed”. When the pulse density is increased to 200 pulses per mm, the foundational densification remains relatively stable, while the nanogratings start to develop in a pulse-to-pulse mechanism. This observation points to a densification process that may precede (from previous pulses) but also accompany the formation of nanogratings as suggested earlier.^20^

On the other hand, IR spectra shift within laser tracks and the densification levels estimated are like those achieved through HPHT treatments. This suggests that the densification mechanism involved a combination of high dynamic pressure and high temperature. Indeed, the observed densification and *ν*^Si−O–Si^_as_ shift cannot be attributed solely to thermal effects, as high-temperature quenching (*i.e.* glass fictive temperature increase) alone cannot achieve high levels of densification in SiO_2_ (typ. limited around 3%).^31,36^ Therefore, the necessity of high pressure becomes evident. However, similarly, the densification level observed cannot be entirely attributed to high pressure. The rapid timescale of femtosecond laser processes implies that without accompanying heat, the pressure alone would not result in significant densification, underscoring the necessity of a combined thermo-mechanical mechanism for the observed changes.

High temperatures facilitate rapid and efficient densification along the laser track, following the laser beam's shape and thermal profile. Densification is not limited to areas around nanopores but extends throughout the laser track, reflecting the energy deposition and the plasma distribution. This process, initiated by a swift temperature increase from light–matter interaction, results in thermal expansion and the generation of a strain wave. Within the type II regime, this strain wave is characterized by a compression wave ahead, fostering densification and a rarefaction wave behind that may trigger the nanocavitation process.^17^

To bridge these observations with a semi-quantitative view of the mechanism, it is essential to estimate the involved pressure. Real-time polariscopic observations have recently confirmed the generation of moderate shockwaves with supersonic velocities, approximately 6 km s^−1^, during fs-laser modifications in silica on a picosecond timescale.^39^ Then a transition to acoustic waves occurs within nanoseconds. At the picosecond scale, stress levels reach the gigapascal range, around 10 GPa, corroborating simulation data.^40^ Using data from the mechanics field, the static high-pressure Hugoniot curve provides a theoretical yet experimentally validated framework to estimate pressure from densification in the silica.^41–44^ Previously, by examining shifts in the main band, we determined a density of approximately 2.5. Employing the Hugoniot curve, this translates to an estimated pressure of around 7 GPa. On the other hand, drawing on correlations between densification ratio and shock wave pressure,^45^ the densification ratio of approximately 13.6% translates into an estimated pressure of around 14 GPa. Meanwhile, recent investigations, through comparisons of Raman spectra of densified silica by shock waves generated by two spatially separated focused fs-beams with compressive hydrostatic loading experiments, have estimated the development of dynamic strains with pressures around 13–15 GPa shortly after irradiation.^23^ These overall estimations, ranging from 7 to 14 GPa, highlight the interplay between high pressure and high temperature in driving the densification process of the material within laser tracks resulting in the observed type II fs-modifications.

## 5 Conclusions

In conclusion, our study employing IR s-SNOM and nano-FTIR has unveiled new insights into the nanoscale densification mechanisms within femtosecond laser-induced nanogratings in silica glass. We have demonstrated the first direct evidence of nanoscale densification, both characterized by a significant shift in the Si–O–Si asymmetric stretching band and the corresponding amplitude and phase contrast around 1130 cm^−1^. We estimated densities inside the laser tracks of approximately 2.4–2.5. This demonstrates a densification “bed” behind the nanogratings, suggesting that the underlying mechanism for glass densification during nanograting formation is quite similar to high temperature high pressure SiO_2_ compression. These insights significantly enrich our understanding of fs-laser-induced nanogratings and pave the way for optimizing their fabrication for specific optical applications.

## Data availability

The data supporting this article have been included as part of the ESI.† Additional data are available upon request from the authors.

## Author contributions

Writing – original draft, N. Shchedrina; writing – review and editing, N. Shchedrina, G. Nemeth, F. Borondics, N. Ollier, M. Lancry; visualization, N. Shchedrina; data curation, N. Shchedrina and G. Nemeth; formal analysis, N. Shchedrina; investigation, N. Shchedrina, G. Nemeth, N. Ollier, and M. Lancry; validation, G. Nemeth, F. Borondics, N. Ollier, and M. Lancry; methodology, G. Nemeth and M. Lancry; resources, F. Borondics and M. Lancry; supervision, N. Ollier and M. Lancry; conceptualization, M. Lancry; funding acquisition, M. Lancry. All authors have read and agreed to the published version of the manuscript.

## Conflicts of interest

There are no conflicts to declare.

## Supplementary Material

NA-OLF-D4NA00409D-s001
