# Supplementary material for: Nano-FTIR spectroscopy reveals SiO2 densification within fs-laser induced nanogratings
Source: Nanoscale Adv. 2024 Aug 6;6(20):5164–70. doi: 10.1039/d4na00409d (PMC11376039; doi:10.1039/d4na00409d)
Supplement: NA-006-D4NA00409D-s001 [file NA-006-D4NA00409D-s001.pdf]

Supplementary Information (SI) for Nanoscale Advances.
This journal is © The Royal Society of Chemistry 2024

Supplementary Information

Nano-FTIR spectroscopy reveals SiO_2 densification within fs-laser induced nanogratings

Shchedrina N.*[1,2], Nemeth G.[3], Borondics F.[3], Ollier N.[2], Lancry M.[1]

1 Institut de Chimie Moléculaire et des Matériaux d'Orsay, Université Paris-Saclay, Rue du doyen Georges Poitou, 91405 Orsay cedex, France

2 Laboratoire des Solides Irradiés Ecole Polytechnique, CNRS, CEA\DRF\IRAMIS, Institut Polytechnique de Paris, 91128 Palaiseau Cedex, France

3 SMIS Beamline, SOLEIL Synchrotron, L'Orme des Merisiers, 91190 Saint Aubin, France

* Corresponding author: nadezhda.shchedrina@universite-paris-saclay.fr

Abstract

This study explores the structural transformations induced by femtosecond (fs) laser inscriptions in glass, with a focus on Type II modifications (so called nanogratings), crucial for advanced optical and photonic technologies. Our novel approach employs scattering-type scanning near-field optical microscopy (s-SNOM) and the synchrotron radiation nanoscale Fourier-transform infrared spectroscopy (nano-FTIR) to directly assess the nanoscale structural changes in the laser tracks, potentially offering a comprehensive understanding of the underlying densification mechanisms. The results reveal the first direct nanoscale evidence of densification driven by HP-HT within fs-laser inscribed tracks, characterized by a significant shift of the main infrared (IR) vibrational structural band of silica glass. It reveals moreover a complex interplay between Type I and Type II modifications.

Keywords: fs-laser writing, type II modifications, nanogratings, s-SNOM, Nano-FTIR spectroscopy, silica glass densification

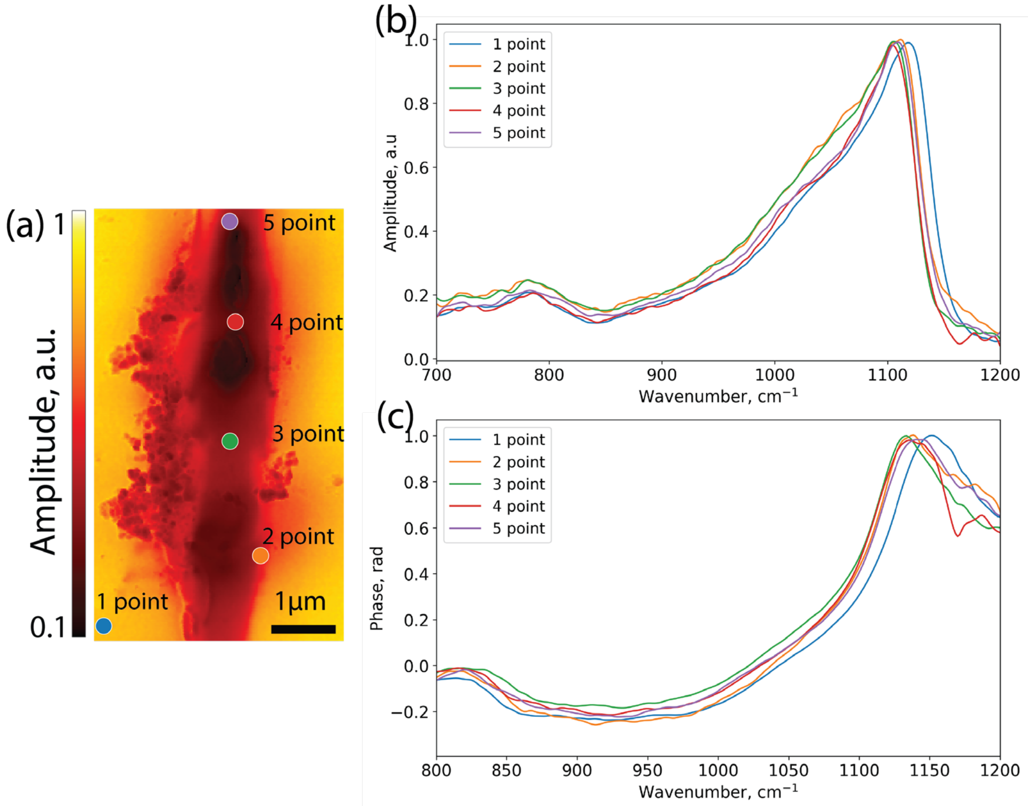

Fig. SI_1. The laser track written with 2 pulses per micron: (a) near-field amplitude map of the Si-O-Si asymmetric stretching mode at 1130 cm^{-1}; (b) synchrotron nano-FTIR amplitude and (c) phase spectra for measured points of pristine material (point 1) and irradiated area inside of the laser track (points 2-5).

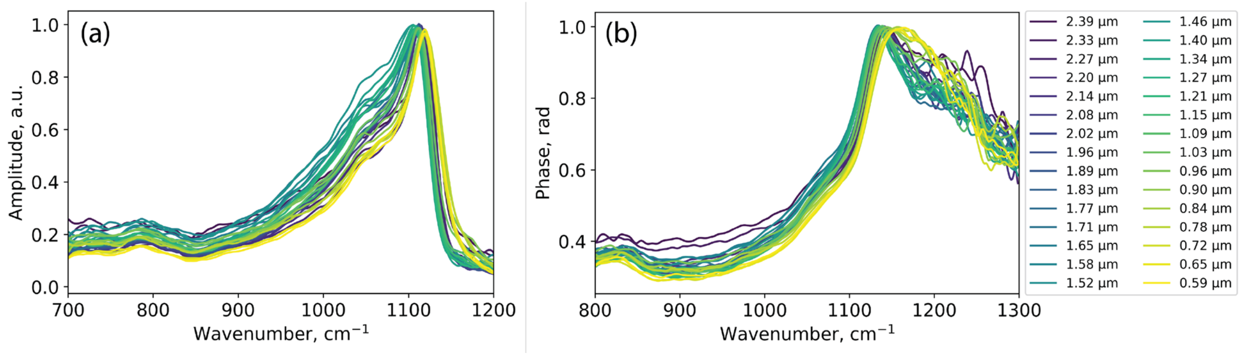

Fig. SI_2. Synchrotron nano-FTIR (a) amplitude and (b) phase spectra obtained along the line profile of the laser track written with 2 pulses per micron. This corresponds to the early birth of nanogratings. The legend indicates the X position of the spectrum on the line profile, as

referenced in Fig. 3a-d of the manuscript. To avoid overloading the graph, only every second spectrum is plotted.

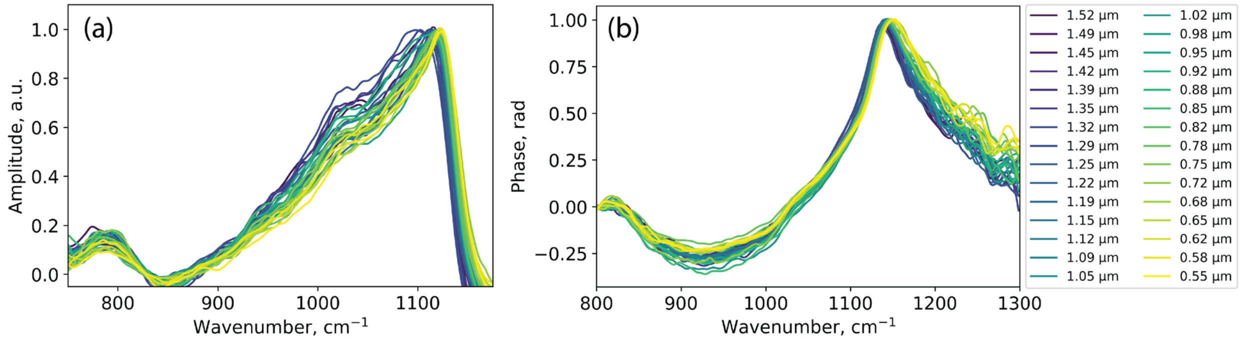

Fig. SI_3. Synchrotron nano-FTIR (a) amplitude and (b) phase spectra obtained along the line profile of the laser track written with 200 pulses per micron. This corresponds to well established nanogratings with a regular arrangement. The legend indicates the X position of the spectrum on the line profile, as referenced in Fig. 4 of the manuscript. To avoid overloading the graph, only every second spectrum is plotted.
